# Decreased vitamin C uptake mediated by *SLC2A3* promotes leukaemia progression and impedes TET2 restoration

**DOI:** 10.1038/s41416-020-0788-8

**Published:** 2020-03-16

**Authors:** Jun Liu, Junshik Hong, Heejoo Han, Jihyun Park, Dongchan Kim, Hyejoo Park, Myunggon Ko, Youngil Koh, Dong-Yeop Shin, Sung-Soo Yoon

**Affiliations:** 1 Cancer Research Institute, Seoul National University College of Medicine, Seoul, Republic of Korea; 2 Department of Internal Medicine, Seoul National University Hospital, Seoul National University College of Medicine, Seoul, Republic of Korea; 3 Biomedical Research Institute, Seoul National University College of Medicine, Seoul, Republic of Korea; 4 School of Life Sciences, Ulsan National Institute of Science and Technology, Ulsan, Republic of Korea

**Keywords:** Cancer metabolism, Acute myeloid leukaemia

## Abstract

**Background:**

Vitamin C suppresses leukaemogenesis by modulating Tet methylcytosine dioxygenase (TET) activity. However, its beneficial effect in the treatment of patients with acute myeloid leukaemia (AML) remains controversial. In this study, we aimed to identify a potential predictive biomarker for vitamin C treatment in AML.

**Methods:**

Gene expression patterns and their relevance to the survival of AML patients were analysed with The Cancer Genome Atlas (TCGA) and Therapeutically Applicable Research to Generate Effective Treatments (TARGET) database cases. In vitro experiments were performed on AML cell lines, a *SLC2A3*-knockdown cell line and patient-derived primary AML cells.

**Results:**

*SLC2A3* expression was significantly decreased in leukaemic blast cells. Below-median *SLC2A3* expression was associated with poor overall survival. Low *SLC2A3* expression was associated with less effective demethylation, and a diminished vitamin C effect in the AML and lymphoma cell lines. *SLC2A3* knockdown in the KG-1 cell line decreased the response of vitamin C. In patient-derived primary AML cells, vitamin C only restored TET2 activity when *SLC2A3* was expressed.

**Conclusion:**

*SLC2A3* could be used as a potential biomarker to predict the effect of vitamin C treatment in AML.

## Background

Recent studies have suggested that vitamin C is a novel metabolic tumour suppressor involved in epigenetic remodelling, and have highlighted vitamin C as a potential innovative treatment strategy for leukaemia.^1–3^ Vitamin C is a cofactor for the enzyme Tet methylcytosine dioxygenase (TET), which regulates the modification of methyl groups in DNA, a regulatory mechanism that has been reported to alter gene expression.^4^ Specifically, TET catalysis is an intermediate step in DNA demethylation, converting the molecule 5-methylcytosine (5-mC) to 5-hydroxymethylcytosine (5-hmC).^5^ TET2 is one of the most frequently mutated genes in haematopoietic malignancies. Somatic deletions and loss-of-function mutations in TET2 are observed in ~10% of de novo acute myeloid leukaemia (AML) patients, ~30% of myelodysplastic syndrome patients and almost 50% of chronic myelomonocytic leukaemia patients.^6–8^ While TET2 mutations are thought to be driver mutations in haematopoietic malignancies, low TET1 levels in hematopoietic and breast cancers correlate with an advanced disease, metastases and poor patient survival.^9,10^ In addition, both TET2 and TET3 are expressed at high levels in murine haematopoietic stem/progenitor cells (HSPCs), and their individual deletion in mice results in aberrant haematopoiesis.^10^ Taken together, a loss of TET function results in aggressive myeloid cancer. In addition, Blaschke et al. also reported that the effects of vitamin C depend on TET, and are mediated by TET1, TET2 and TET3.^11^

Vitamin C is transported across the cellular membrane by sodium vitamin C cotransporters (SVCTs) and glucose transporters (GLUTs); the latter are encoded by the *SLC2A* family.^12^ While SVCTs transport the reduced form of vitamin C into the cell, GLUTs (mainly GLUT1 and GLUT3) transport the oxidised form of vitamin C, dehydroascorbate (DHA). After import, DHA is reduced to vitamin C at the expense of glutathione, thioredoxin and nicotinamide adenine dinucleotide phosphate (NADPH).^13^ For the haematological system, SVCT1 (*SLC23A1*) is not mainly expressed by haematopoietic cells.^14,15^ SVCT2 is expressed in most haematopoietic cells, but is expressed at 14-fold higher levels in haematopoietic stem cells and multipotent progenitors (HSCs/MPPs) compared with restricted haematopoietic progenitors.^2^ Based on this phenomenon, maintaining a relatively higher level of vitamin C was essential to sustain the normal functions of HSCs/MPPs. However, malignant cells (e.g. the AML cell line HL-60) lack the capacity to transport the reduced form of vitamin C, ascorbic acid, but show an ability to transport DHA, the oxidised form of vitamin C.^16^

Although recent reports have suggested that vitamin C could be a novel metabolic tumour suppressor, the results regarding the clinical beneficial effect of vitamin C remain controversial, although patients suffering from haematological diseases often display low vitamin C levels.^17,18^ In the current study, we aimed to identify biomarkers that can predict the effect of vitamin C treatment in AML cells.

## Methods

### Microarray data analysis

For the gene expression datasets GSE37307 and GSE9476, raw data (matrix file) were downloaded from the NCBI Gene Expression Omnibus website (http://www.ncbi.nlm.nih.gov/geo/). These data were normalised using the normalizeBetweenArrays module in the limma package (https://bioconductor.org/packages/release/bioc/html/limma.html).^19,20^ All the analyses were performed using the R (version 2.15.0, www.r-project.org) and Bioconductor packages.^21^

### Cell lines and cell culture

Four AML cell lines, OCI-AML3, HEL, KG-1 and HL-60, and two diffuse large B-cell lymphoma (DLBCL) cell lines, OCI-LY1 and OCI-LY19, were purchased from DSMZ (Leibniz Institute DSMZ-German Collection of Microorganisms and Cell Cultures). One DLBCL cell line, TOLEDO, was purchased from the American Type Culture Collection (ATCC). HEL, KG-1 and HL-60 cells were cultured in RPMI 1640 medium supplemented with 10% foetal bovine serum (FBS), l-glutamine and 1% penicillin–streptomycin (Gibco, Grand Island, NY, USA) at 37 °C and 5% CO_2_. OCI-AML3 and OCI-LY19 cells were cultured in α-MEM supplemented with 20% FBS, l-glutamine and 1% penicillin–streptomycin (Gibco) at 37 °C in the presence of 5% CO_2_. OCI-LY1 cells were cultured in Iscove Modified Dulbecco Media (IMDM) supplemented with 20% FBS, l-glutamine and 1% penicillin–streptomycin (Gibco, Grand Island, NY, USA) at 37 °C and 5% CO_2_.

### Patient-derived primary AML CD34+ blast cells and cell culture

We used seven patient-derived primary AML cells. The origin of the primary leukaemia cells was cryopreserved leukaemia cells in peripheral blood collected through therapeutic leukapheresis. A CD34 microbead kit (130-046-703) was purchased from MACS Miltenyi Biotec (Bergisch Gladbach, Germany). CD34+ cells were sorted by magnetic-activated cell sorting (MACS) and cultured ex vivo at 2 × 10^6^/mL with StemSpan Serum-Free Expansion Medium (SFEM) supplemented with 100 μg/mL SCF, 50 μg/mL FLT3L, 25 μg/mL GCSF and 25 μg/mL IL-3 at 37 °C and 5% CO_2_.

The use of the patient-derived primary AML cells from seven patients was reviewed and approved by the Institutional Review Board of the Seoul National University Hospital (Approval number: H-1905-133-1035).

### Cell viability assay

As described previously,^22^ cells were seeded into 96-well plates, cultured for 24 h and then exposed to increasing doses of vitamin C for 48 h. Next, 10 μL of Cell Counting Kit 8 (CCK8) reagent (Dojindo, Japan) was added to each well and incubated for 4 h. Then, the optical density (OD) values were measured at 450 nm on a microplate reader.

### Knockdown of SLC2A3 expression by RNA interference in AML cells

The GLUT3 gene *SLC2A3* was knocked down in the KG-1 AML cell line using RNA interference (RNAi) technology. KG-1 cells were transfected with a set of small-interfering RNAs (siRNAs) targeting *SLC2A3* or a non-targeting control siRNA. Transfections were conducted using electroporation with the LONZA SF kit FF-100 with 4D X units. siRNA sequences used in this study are shown in Table S1.

### Antibodies and reagents

l-Ascorbic acid was purchased from Sigma-Aldrich (Sigma-Aldrich, St. Louis, MO, USA, A4544-25G). Primary antibodies, including anti-GLUT3, anti-5-mC and anti-5-hmC, were purchased from Abcam (Abcam, Cambridge, MA, USA). Anti-TET2 was purchased from Cell Signaling Technology (CST, Beverly, MA, USA).

### Western blot analysis

As described previously,^22^ whole-cell lysates were collected using Kinexus protein lysis buffer [containing 20 mM MOPS (pH 7.0), 2 mM EGTA, 5 mM EDTA, 30 mM sodium fluoride, 60 mM β-glycerophosphate (pH 7.2), 20 mM sodium pyrophosphate, 1 mM sodium orthovanadate, 1% Triton X-100, 1 mM PMSF and 1 g/mL protein inhibitor cocktail (Hoffmann-La Roche, Ltd., Switzerland)]. The cell lysates were separated on 8–15% SDS-PAGE gels, transferred onto nitrocellulose membranes, blocked, washed and incubated overnight at 4 °C with the appropriate primary antibodies. After washing, the membranes were incubated with the appropriate HRP-conjugated secondary antibody, and enhanced chemiluminescence (ECL) was used to develop chemiluminescence.

### Quantitative real-time PCR

The expression of *SLC2A3* was measured using quantitative real-time PCR (qPCR). The 18S rRNA served as the internal control. The assay was conducted using a Bioneer SYBR Green qPCR premix and an Applied Biosystems 7500 PCR machine. Total RNA was isolated with TRIzol™ reagent, and 2.5 ng of total RNA was used to produce cDNA from RNA with cDNA EcoDry Premix (Takara, Kusatsu, Shiga, Japan) according to the manufacturer’s instructions. Primer sequences used in this study are shown in Table S2.

### Fluorescence-activated cell-sorting analysis

A total of 10^6^ cells were used in the fluorescence-activated cell-sorting (FACS) assay for each sample. The cells were resuspended in 200 μL of FACS buffer, and the CD34 antibody was added. The mixture was incubated for 1 h in the dark at 4 °C. After incubation, 500 μL of FACS buffer was added to the tube, and the tube was centrifuged at 3000 rpm for 5 min. The cells were resuspended in 500 μL of FACS buffer and placed on ice.

### ROS measurement

The cells were collected and then incubated with a final concentration of 10 μM DCF-DA (2′, 7′-dichlorofluorescein diacetate) for 30 min. The cells were washed with PBS, resuspended in FACS buffer and analysed using FACS.

### Dot-blot analysis

DNA samples were isolated by proteinase K digestion and phenol/chloroform extraction. DNA was denatured, and twofold serial dilutions were spotted onto a nitrocellulose membrane in an assembled Bio-Dot apparatus (Bio-Rad, Hercules, California, USA). After drying, blocking and washing, the membranes were incubated overnight at 4 °C with the appropriate primary antibodies. Then, the HRP-conjugated secondary antibody was added to the membrane and incubated for 1 h, and ECL was used to develop chemiluminescence. In addition, another membrane was stained with 0.02% methylene blue in 0.3 M sodium acetate (pH 5.2) to visualise the DNA as a total genomic DNA loading control.

### Nuclear extraction

The nuclear extract was prepared using the Nuclear Extraction Kit (Abcam: ab113474). The extraction procedure was performed according to the user manual. The extracted protein was frozen at −80 °C until use.

### Immunoprecipitation

Immunoprecipitation was performed using a Pierce Classic IP kit (Thermo Scientific: 26146). The procedure was performed according to the user manual. The nuclear extracts were used in the immunoprecipitation. The TET2 antibody was used at a 1:100 dilution.

### Quantification of TET2 hydroxylase activity

TET2 hydroxylase activity was measured using a TET Hydroxylase Activity Quantification Kit (Colormetric) (Abcam: ab156912). The procedure was performed according to the user manual. Two micrograms of the TET2 protein were used to quantify the activity.

### Ascorbic acid assay

In this experiment, 5 × 10^6^ cells were used to measure the ascorbic acid levels. The cell pellet was lysed with a pre-lysis buffer (Abcam: ab113474). Then, the ascorbic acid level in the supernatant was measured using the method described in the user manual.

### Statistical analysis

Data are presented as the mean ± standard deviation (SD). The samples from each group were compared by Student’s *t* test, and multiple comparisons between groups were performed using analysis of variance (ANOVA). The difference in survival time was assessed using the log-rank test. Analyses were conducted with SPSS version 19.0.1. A meta-analysis of survival data was performed using the GraphPad Prism program.

## Results

### SLC2A3 gene expression was significantly decreased in AML cells compared with normal haematopoietic cells

**Fig. 1:**
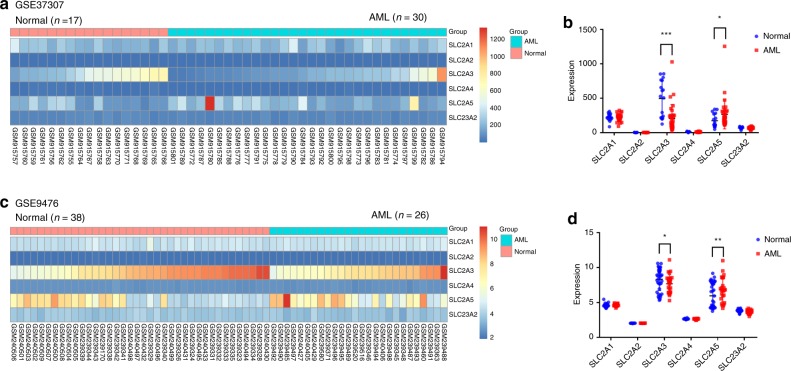
*SLC2A3* gene expression was significantly decreased in blast cells compared with normal haematopoietic cells. The expression of *SLC2A1*, *SLC2A2*, *SLC2A3*, *SLC2A4*, *SLC2A5* and *SLC23A2* between normal haematopoietic cells from healthy controls and AML blast cells from patients, respectively. The data were obtained from a public microarray database. Error bars represent the mean ± standard error of the mean (SEM). *p < 0.05, **p < 0.01 (Student’s *t* test). **a** Heat map of the GSE37307 dataset. **b** Dot plot of the normalised expression in the GSE37307 dataset. **c** Heat map of the GSE9476 dataset. **d** Dot plot of the normalised expression in the GSE9476 dataset.

Based on the above knowledge, our question was whether SVCT, GLUT or their encoding genes (*SLC23A2*, *SLC2A1* and *SLC2A3*), were downregulated in primary AML blast cells from AML patients. We analysed the gene expression patterns of *SLC23A2* and the major GLUT family genes in primary AML blast cells, using previously published microarray datasets (GSE37307 and GSE9476), and determined that *SLC2A3* gene expression was significantly decreased in blast cells compared with normal haematopoietic cells. The fold change in *SLC2A3* expression in the GSE9476 dataset (AML/Normal) was 0.90, and the *p* value was <0.05. The fold change in the GSE37307 dataset (AML/Normal) was 0.32 (*P* < 0.01). In contrast, the expression of *SLC2A1*, *SLC2A2* and *SLC23A2* was not significantly different between primary AML cells and normal haematopoietic cells (Fig. 1).

### Below-median SLC2A3 expression was associated with poor overall survival

**Fig. 2:**
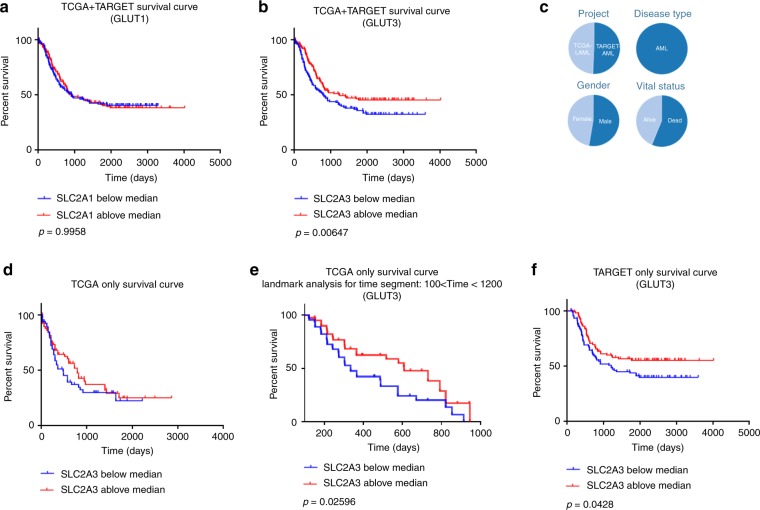
Below-median *SLC2A3* expression was associated with poor overall survival. **a** Overall survival of TCGA + TARGET patients with AML stratified according to the GLUT1 expression level. **b** Overall survival of TCGA + TARGET patients with AML stratified according to the GLUT3 expression level. **c** Characteristics of the 338 analysed cases from TCGA and the TARGET databases. **d** Overall survival of TCGA patients with AML stratified according to the GLUT3 expression level. **e** Overall survival of TARGET patients with AML stratified according to the GLUT3 expression level.

The overall survival of the patients was analysed to further evaluate the role of GLUT3. We analysed the relevance of *SLC2A3* expression to the overall survival of AML patients in 338 cases from The Cancer Genome Atlas (TCGA) and the Therapeutically Applicable Research to Generate Effective Treatments (TARGET) database. Among those cases, patients were divided into two groups: those with above-median *SLC2A3* expression and those with below-median *SLC2A3* expression. Below-median *SLC2A3* expression was associated with a poor overall survival, whereas the GLUT1 expression level showed no correlation with the overall survival rate (Fig. 2a–c). These two gene expression datasets were obtained from adults and paediatric patients, respectively. The overall age range of the patients included in these two datasets was 0.02 (8 days) to 88 years old. The age range of the patients in the TARGET dataset was 0.02 (8 days) to 29 years, and the age range of the patients in the TCGA was 18–88 years. If we only used the TCGA dataset, the median survival for the groups displaying a survival below and above the median value was 486 and 792 days, respectively (Fig. 2d). Because the survival curve crossed at the starting and ending regions, the landmark method was used to avoid guarantee-time bias. Below-median *SLC2A3* expression group showed significantly inferior median overall survival compared with those with above-median expression group (*P* = 0.02597 by landmark analysis, Fig. 2e). If we only use the TARGET dataset, below-median *SLC2A3* expression group also showed significantly inferior median overall survival compared with their counterpart (*P* = 0.0428 by log-rank test).

### Expression of GLUT3 and its effect on vitamin C

**Fig. 3:**
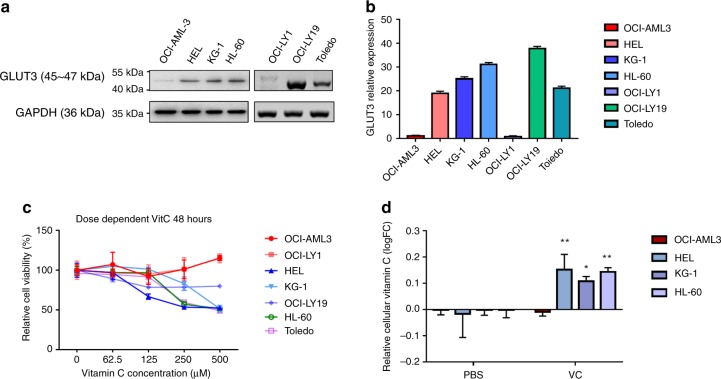
Decreased expression of *SLC2A3* influences the effect of vitamin C. **a** GLUT3 expression was measured in four AML cell lines and three DLBCL cell lines using western blotting. **b** The GLUT3 mRNA level was measured in four AML cell lines and three DLBCL cell lines using qPCR. RNA from OCI-AML3 cells served as the control to compare the relative expression in AML cell lines, and RNA from OCI-LY1 cells serves as the control to compare the relative expression in DLBCL cell lines. **c** The proliferation-suppressing effect of vitamin C was determined by performing cell viability assays. Four AML cell lines and three DLBCL cell lines were treated with increasing doses of L-AA (up to 500 μM) for 48 h. **d** The cytoplasmic vitamin C level was measured using the ascorbic acid assay kit; the cells were treated with 250 μM L-AA for 48 h, *p < 0.05, **p < 0.01 (two-way ANNOVA).

We examined the GLUT3 expression level in four AML cell lines and three DLBCL cell lines. GLUT3 expression was significantly decreased in the OCI-AML-3 and OCI-LY1 cell lines (Fig. 3a). The level of the GLUT3 mRNA was also detected in these seven cell lines using qPCR. Consistent with the protein expression, the mRNA level was significantly decreased in the OCI-AML-3 and OCI-LY1 cells (Fig. 3b). To determine whether the decreased expression of *SLC2A3* would affect vitamin C, we treated the seven cell lines with increasing doses of vitamin C (up to 500 μM) for 48 h. The seeding density was 10^4^ cells/well (200 μL). The vitamin C treatment was performed using L-AA. Based on the results of the cell viability assay, the OCI-AML-3 and OCI-LY1 cells did not respond to vitamin C; however, the proliferation of NB4, HEL, HL-60, OCI-LY19 and Toledo cells was suppressed by 250 μM vitamin C (Fig. 3c and Table S3). We postulated that the responding and non-responding samples displayed different vitamin C uptake levels. Cytoplasmic vitamin C levels in four AML cell lines were measured using the ascorbic acid assay to confirm this hypothesis. Vitamin C uptake was significantly increased in HEL, KG-1 and HL-60 cells, but not in OCI-AML3 cells (Fig. 3d).

### Low expression of GLUT3 impedes the restoration of TET2 activity

**Fig. 4:**
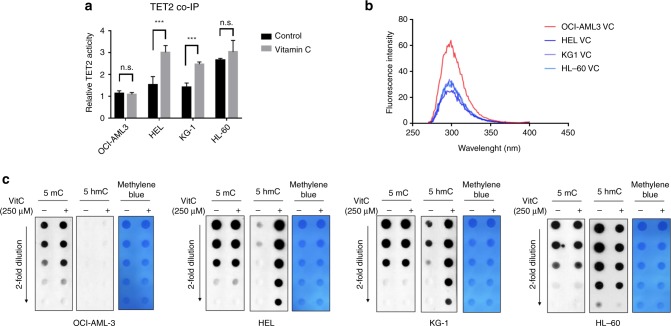
Low expression of *SLC2A3* impedes TET2 restoration. **a** TET2 hydroxylase activity was quantified using a TET activity assay kit after cells were treated with 250 μM vitamin C for 48 h; 2 μg of TET2 protein was purified using co-IP, *p < 0.05, **p < 0.01 (two-way ANOVA). **b** Fluorescence quenching of the TET2 caused by vitamin C. **c** DNA dot blots showing 5-mC and 5-hmC levels in the genomic DNA from four AML cell lines treated with PBS or 250 μM vitamin C for 48 h.

Thus, we hypothesised that reduced vitamin C uptake mediated by GLUT3 would impede the restoration of TET2 activity. We used the TET hydroxylase activity quantification kit to quantify the activity of the TET2 enzyme in these cell lines. After cells were treated with 250 μM vitamin C for 48 h, the TET2 protein was immediately purified using a co-immunoprecipitation (co-IP) with a TET2 antibody. Then, 2 μg of the TET2 protein were used to measure the TET2 activity. As shown in our data, treatment of the four cell lines with 250 μM vitamin C for 48 h revealed a significant increase in TET2 activity in HEL and KG-1 cells (Fig. 4a). The TET2 C-terminal catalytic domain (CD) contains 5 tryptophan (W) and 35 tyrosine (Y) residues,^23^ which are known to emit intrinsic fluorescence (maximum emission: 303 nm).^24^ Vitamin C can directly bind to the CD domain of TET proteins, and quenches the intrinsic fluorescence.^4^ As shown in our data, TET2 proteins, which were extracted from HEL, KG-1 and HL-60 cells, showed efficiently quenched fluorescence intensity compared with OCI-AML3 (Fig. 4b). This indicated that in HEL, KG-1 and HL-60 contained vitamin C bonded with TET2, but there was no vitamin C-bonded TET2 in OCI-AML3. In addition, consistent with the TET2 activity, the level of 5-hmC in the genomic DNA was also increased in HEL and KG-1 cells, but not in OCI-AML3 and HL-60 cells (Fig. 4c).

In addition to its effects on TET2, vitamin C mainly functions as an antioxidant. Therefore, the levels of reactive oxygen species (ROS) were measured using DCF-DA. The four AML cell lines were treated with PBS or 250 μM L-AA for 48 h, and then the ROS level was measured using DCF-DA. As shown in Fig. S1, the ROS levels were decreased in OCI-AML3 and HEL cells, but no change was detected in KG-1 and HL-60 cells. By comparing the ROS data with the results of the cell viability assay, the antioxidant function of vitamin C was not the major cause of the inhibition of proliferation.

### Knockdown of the SLC2A3 gene impedes the response to vitamin C

**Fig. 5:**
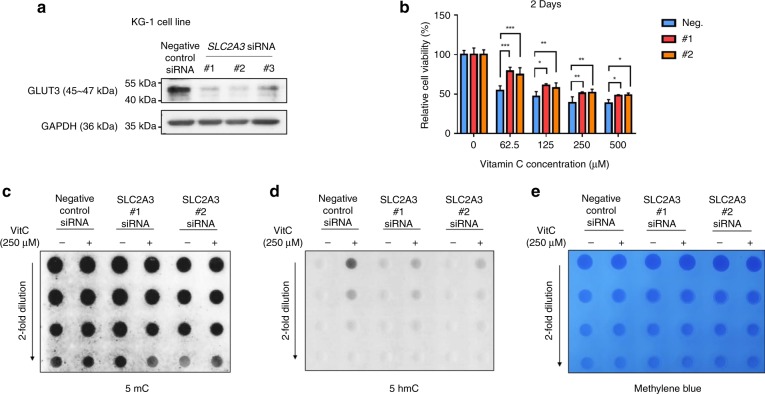
Knockdown of the *SLC2A3* gene impedes the response to vitamin C. **a** Twenty-four hours after siRNA transfection in KG-1 cell lines, GLUT3 expression was measured using a western blot analysis. **b** After siRNA transfection in KG-1 cell lines, the proliferation-suppressive effect of vitamin C was detected by cell viability assays. The transfected cell lines were treated with increasing doses of vitamin C (up to 500 μM) for 48 h, *p < 0.05, **p < 0.01 (two-way ANOVA). **c**–**e** DNA dot blots showing 5-mC and 5-hmC levels in the genomic DNA from transfected KG-1 cell lines treated with PBS or 250 μM vitamin C for 48 h. Methylene blue staining was performed as a loading control.

We knocked down the *SLC2A3* gene in KG-1 cells using the RNAi technique. The siRNAs #1 and #2 showed a good knockdown effect of GLUT3 (Fig. 5a); therefore, these two siRNAs were used in the following experiments. According to the results of the cell viability assays, knockdown of the *SLC2A3* gene resulted in a reduced effectiveness of vitamin C after treatment for 48 h (Fig. 5b). Knockdown of the *SLC2A3* gene also significantly abolished the vitamin C-induced increase in 5-hmC levels in genomic DNA, as detected with the dot-blot assay (Fig. 5c–e).

### Role of SLC2A3 in patient-derived primary leukaemia cells

**Fig. 6:**
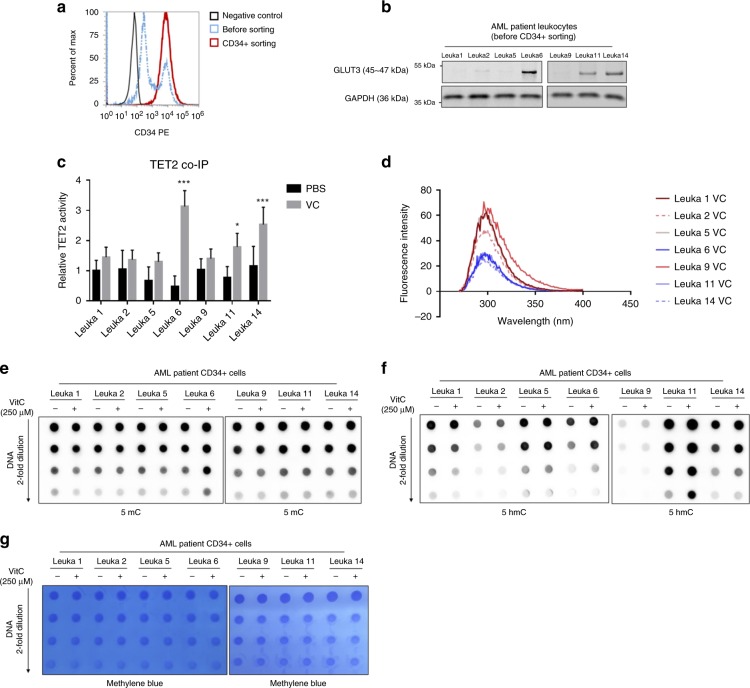
Role of *SLC2A3* in patient-derived primary leukaemia cells. **a** The sorting effect was detected by a FACS analysis of CD34+ cells; representative data are shown here. **b** The GLUT3 expression levels in patient-derived primary AML cells were measured using a western blot analysis. **c** TET2 activity was measured in patient-derived primary AML cells before or after treatment with 250 μM vitamin C for 48 h; 2 μg of TET2 protein was purified using co-IP, *p < 0.05, **p < 0.01 (two-way ANOVA). **d** Fluorescence quenching of the TET2 caused by vitamin C. **e**–**g** DNA dot blots showing the levels of 5-mC and 5-hmC in the genomic DNA from patient-derived AML cells treated with PBS or 250 μM vitamin C for 48 h. Methylene blue staining was performed as a loading control.

We used seven patient-derived primary leukaemia cells to confirm the role of *SLC2A3* in vitamin C treatment. The sorting effectiveness of CD34+ cells was determined by FACS (Fig. 6a). GLUT3 levels were detected using a western blot analysis (Fig. 6b). After cells were treated with 250 μM vitamin C, the TET2 protein was immediately purified using a co-IP with a TET2 antibody. Then, 2 μg of the TET2 protein were used to measure the TET2 activity. TET2 activity was significantly restored in Leuka6, Leuka11 and Leuka14 cells (Fig. 6c). In addition, consistent with TET2 activity, fluorescence intensity scan showed that TET2 from Leuka 6, 11 and 14 efficacies quenched the instinct fluorescence caused by binding with vitamin C. However, other TET2 proteins did not bind with vitamin C even though vitamin C was treated. In addition, the dot blot also showed a significant increase in 5-hmC levels in Leuka6, Leuka11 and Leuka14 cells (Fig. 6d–f).

## Discussion

Over the past century, the notion that vitamin C can be used to treat cancer has generated much controversy.^25^ Recently, Zhao et al. showed that vitamin C with decitabine activates TET2 in leukaemia cells, and significantly improves overall survival in elderly patients. In 2017, Agathocleous et al. and Cimmino et al. reported that vitamin C suppressed haematological malignancies by modulating TET2 activity.^1–3^ However, the question that remains is what dosage of vitamin C is ultimately taken up by malignant cells? In the current study, we found that GLUT3, encoded by the *SLC2A3* gene, prompted malignant cells to uptake vitamin C. In AML patient blast cells, GLUT3 expression tends to be reduced, which would impede vitamin C uptake and abolish its clinical benefit.

Although both GLUT1 and GLUT3 are able to transport vitamin C, our data show that GLUT3 is the major transporter for vitamin C in AML cells. As shown in the study by Beltran et al., the knockdown of endogenous GLUT3 expression is sufficient to completely abolish the effect of ascorbic acid.^26^ In addition, overexpression of GLUT1 is associated with chemoresistance and an inferior overall survival, which indirectly suggests that expression of GLUT1 is not beneficial compared with the expression of GLUT3.^27^ Taken together, GLUT1 was not able to compensate for and transport vitamin C to enhance TET activity in AML cells.

The TET enzyme can catalyse the iterative oxidation of 5-mC to 5-hmC, 5-formylcytosine (5fC) and 5-carboxylcytosine (5caC). These oxidised mCs are key intermediates in DNA demethylation via replication-dependent dilution or base-excision repair (BER).^28^ In the current study, we found that vitamin C led to a striking global increase in 5-hmC by dot-blot analysis when GLUT3 was expressed. In contrast, the global levels of 5-mC were not altered. A similar phenomenon in which vitamin C increases global 5-hmC without affecting 5-mC has also been reported by Blaschke et al.^11^. In addition to its role as an intermediate in DNA demethylation, 5-hmC was recently reported to have a specific gene-regulatory function,^29^ and our results support the role of 5-hmC. Moreover, the original level of 5-hmC in HL-60 cells was extremely high, and TET function was not altered. Therefore, the 5-hmC level would not increase, despite the treatment with vitamin C. Based on these data, vitamin C may only restore the TET function, but not reverse the aberrantly hyperactive TET function.

The precise mechanism by which vitamin C acts as a cofactor to mediate TET2 restoration has not yet been elucidated. Vitamin C was shown to interact directly with the catalytic domain of TET proteins, and provide a local reducing environment that increases the recycling efficiency of the Fe(II) cofactor.^4,28^ Recently, Xue et al. showed that the green alga *Chlamydomonas reinhardtii* contains a 5-mC-modifying enzyme (CMD1) and a TET homologue, and unlike previously described TET enzymes, which use 2-oxoglutarate as a co-substrate, CMD1 uses vitamin C as an essential co-substrate. Vitamin C donates the glyceryl moiety to 5-mC with the concurrent formation of glyoxylic acid and CO_2_.^30^ Therefore, in humans, vitamin C may not only function as a cofactor of TET, but also as a substrate that directly contributes to DNA modifications.

We are aware of the limitations of the current study. We used limited numbers of cell lines and patient-derived primary AML cells. Our findings should be validated in future studies with a larger number of patients. Nevertheless, we simply and clearly identified the association between GLUT3 and the tumour-suppressive effect of vitamin C. Since the data were retrieved from a database, and only limited clinical information was provided, we estimated the difference in overall survival as an exploratory analysis to justify further evaluations designed to define the role of GLUT3. We are performing ongoing experiments to define the mechanism underlying the decreased expression of GLUT3, and to determine appropriate methods to improve vitamin C-mediated restoration of TET activity by inducing GLUT3 expression in AML cells. In addition, we are planning a clinical trial to evaluate whether *SLC2A3* can be applied as a predictive biomarker for vitamin C treatment in patients with AML. Based on our data, GLUT3 plays important roles in both paediatric and adult patients. However, GLUT3 may play a more critical role in young patients than in older patients (Fig. 2d, e). We did not use cell lines derived from paediatric patients or material from younger patients, and thus the age-related responses should be explored in further studies.

In summary, the expression of the GLUT3 gene was significantly decreased in AML cells compared with normal haematopoietic cells, and low GLUT3 expression was associated with a poor overall survival of patients with AML. The decreased expression of GLUT3 impaired the effect of vitamin C, and the restoration of TET activity in AML cell lines and patient-derived primary AML cells. GLUT3 represents a potential biomarker to predict the effect of vitamin C treatments.

## Supplementary information

Supplemental online material

Leukapheresis_list

## Data Availability

All data and materials generated and/or analysed during the current study are available from the corresponding author upon reasonable request.
