# Supplementary material for: Decreased vitamin C uptake mediated by SLC2A3 promotes leukaemia progression and impedes TET2 restoration
Source: Br J Cancer. 2020 Mar 16;122(10):1445–52. doi: 10.1038/s41416-020-0788-8 (PMC7217885; doi:10.1038/s41416-020-0788-8)
Supplement: Supplementary file 1 — Supplemental online material [file 41416_2020_788_MOESM1_ESM.docx]

**Supplemental online material**

**Decreased vitamin C uptake mediated by SLC2A3 promotes leukaemia progression and impedes TET2 restoration**

**Table S1. siRNA sequences**

| No | siRNA name | Duplex sequence | |
| --- | --- | --- | --- |
| 1 | *SLC2A3*#1 | Sense | 5'-GAGAAGAUCAUAAAGGAAUU-3' |
|  |  | Antisense | 5'-AUUCCUUUAUGACUUCUCUU-3' |
| 2 | *SLC2A3*#2 | Sense | 5'-GCAAUUCAAUGCUGAUUGUUU-3' |
|  |  | Antisense | 5'-ACAAUCAGCAUUGAAUUGCUU-3' |
| 3 | *SLC2A3*#3 | Sense | 5'-GUGGUUAAUACUAUCUUCAUU-3' |
|  |  | Antisense | 5'-UGAAGAUAGUAUUAACCACUU-3' |
| 4 | Negative control | Sense | 5'-CCUCGUGCCGUUCCAUCAGGUAGUU-3' |
|  |  | Antisense | 5'-CUACCUGAUGGAACGGCACGAGGUU-3' |

**Table S2. Primer sequences**

| *SLC2A3*-F | 5’-TTCGTCTCTAGCCTGCACTG-3’ |
| --- | --- |
| *SLC2A3-*R | 5’-ACACAACTTCTCCGGGTGAC-3’ |
| 18s-F | 5’-GAACAACTGCGAAAGCATTTGC-3’ |
| 18s-R | 5’-CCTGGTAAGTTTCCCCGTGTTG-3’ |

**Table S3. IC50 for each cell lines**

| **Cell line** | **IC50 (μM)** |
| --- | --- |
| **OCI-AML-3** | **>500** |
| **OCI-LY1** | **>500** |
| **HEL** | **75.25** |
| **KG-1** | **232.1** |
| **OCI-LY19** | **64.21** |
| **Hl-60** | **189.7** |
| **Toledo** | **148** |

**
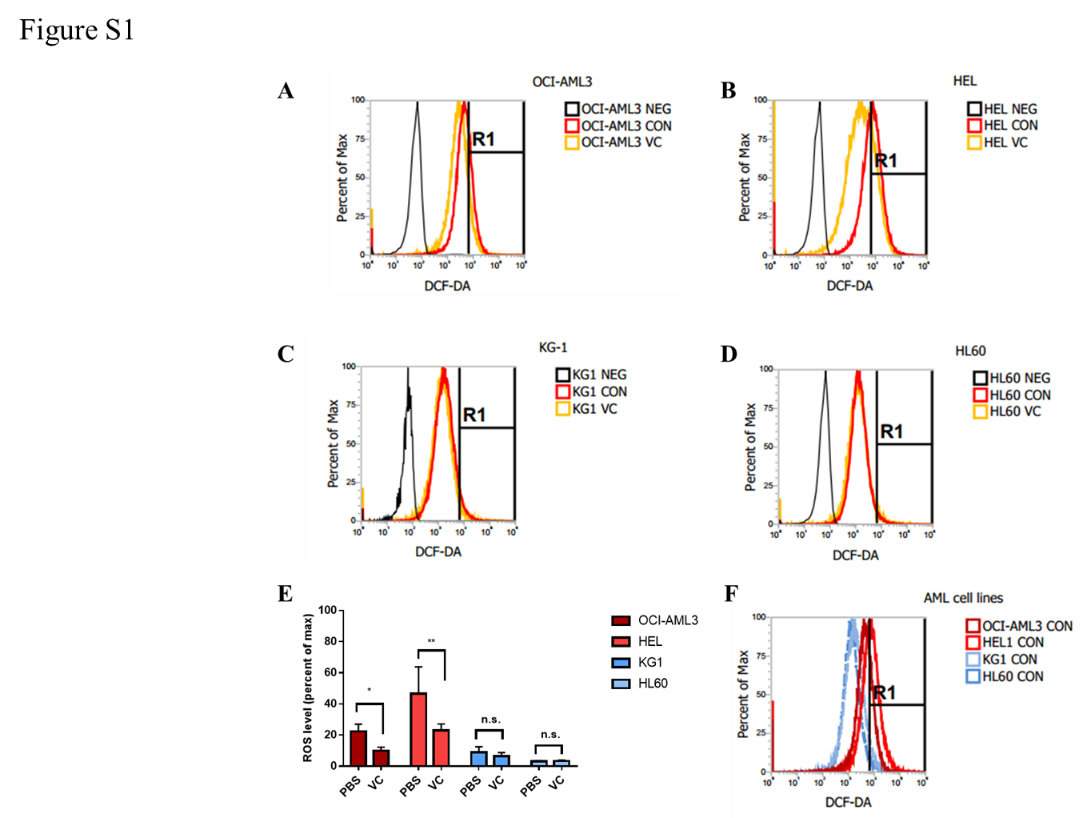
**

**Figure S1. Antioxidative function of vitamin C is not related with the proliferation inhibition**

(A-D) Intracellular ROS levels was measured through DCF-DA based FACS analysis.

(E) Quantification of the ROS levels in 4 AML cell lines.

(F) ROS level overlay of the 4 AML cell lines before treatment.
